# Tocilizumab, an Exploratory Treatment for Severe COVID-19 Patients

**DOI:** 10.1155/2022/6375870

**Published:** 2022-03-15

**Authors:** Yong Wang, Yongfeng Chen, Xiangdong Zhou

**Affiliations:** ^1^Department of Rheumatology, Southwest Hospital, Army Medical University, Chongqing 400038, China; ^2^Taikang Tongji COVID-19 Hospital, Wuhan 430000, China; ^3^Department of Respiratory and Critical Care Medicine, Southwest Hospital, Army Medical University, Chongqing 400038, China

## Abstract

The coronavirus disease 2019 (COVID-19) may cause cytokine storm and respiratory illness such as pneumonia and progressive respiratory failure. Tocilizumab (TCZ), a monoclonal antibody that targets the interleukin-6 (IL-6) receptor, was approved as an alternative treatment for severe COVID-19 patients despite limited real-world clinical data in China. In the present study, we will discuss and evaluate the treatment response of TCZ therapy in patients with COVID-19. The clinical characteristics, treatment, laboratory parameters of IL-6, C-reactive protein (CRP), lymphocyte counts before and after TCZ therapy, and clinical outcomes in the 13 patients with COVID-19 were retrospectively evaluated according to the related medical records. The results showed that 13 patients with COVID-19 were totally included in this study. One of them was moderately ill, 8 were seriously ill, and 4 were critically ill. Eleven patients received TCZ administration once, while the other 2 patients received it twice. The median level of IL-6 before TCZ administration was 27.91 (7.42–210.90) pg/mL. Serum IL-6 level tended to further spike firstly and then gradually decreased after TCZ therapy in 10 patients. A persistent and dramatic increase of IL-6 was observed in 2 patients who were finally dead. The CRP levels of 76.92% (10/13) of the patients were above the normal range before the start of TCZ therapy and gradually declined after the TCZ treatment. No. 1 and No. 10 patients finally died accompanied by the corresponding lymphocyte counts persistently dropping. No. 13 patient became exacerbated possibly due to inducing severe bacterial infection after TCZ treatment, while the other 10 patients showed clinical improvement. In summary, the study revealed that TCZ may have a certain therapeutic effect on severe COVID-19 patients with a risk of the cytokine storm. It is necessary to further evaluate the efficacy and safety of TCZ by rigorous randomized controlled trial in the next step.

## 1. Introduction

Since December 2019, an outbreak of a novel coronavirus disease 2019 (COVID-19) was reported in Wuhan, China, which has subsequently affected more than 200 countries, areas, or territories worldwide. Most patients with COVID-19 exhibit mild to moderate symptoms, but approximately 15% progress to severe pneumonia, and about 5% eventually develop acute respiratory distress syndrome, septic shock, and/or multiple organ failure [1, 2]. These severe COVID-19 patients show substantially elevated serum levels of proinflammatory cytokines, including interleukin-6 (IL-6) and IL-2, as well as IL-1, IL-8, and tumor necrosis factor, characterized as cytokine storm, which may lead to respiratory failure or even death [3–5]. Therefore, early identification, treatment, and prevention of the cytokine storm are of crucial importance for the patients.

The proinflammatory IL-6 appears as one of the key cytokines leading to the inflammatory storm, which may result in increased alveolar-capillary blood-gas exchange dysfunction, which seems to have a prominent role in this inflammatory cascade [3–5]. One clinical trial (https://clinicaltrials.gov/ct2/show/ChiCTR2000029765), using the IL-6 receptor-targeted monoclonal antibody tocilizumab (TCZ), which blocks IL-6-mediated signals by inhibiting IL-6 binding to transmembrane and soluble IL-6 receptors, reported quick control of fever and an improvement of respiratory function in 21 patients with severe COVID-19 treated in Anhui, China [6]. Therefore, TCZ is recommended in seriously ill patients with elevated IL-6 by the Diagnosis and Treatment Protocol for COVID-19 (Trial Version 7) issued by the National Health Commission of China [7]. In the retrospective observational study, all 21 patients, including seventeen seriously ill and four critically ill, had been discharged on average 15.1 days, and no patient became exacerbated or died after taking TCZ, indicating that this drug had almost a magical therapeutic effect on severe and critical COVID-19 patients [8]. However, a meta-analysis showed that there was insufficient evidence regarding the clinical efficacy and safety of TCZ in patients with COVID-19 [9]. Therefore, there are still limited real-world data about the effect of TCZ on inflammatory activity in COVID-19 patients [10].

In the retrospective observational study, we will present the therapeutic response of TCZ in these 13 patients with COVID-19 and provide some experience for the clinical application.

## 2. Methods

### 2.1. Study Design and Participants

The patients infected with COVID-19, who were treated with TCZ from February 29 to March 7, 2020, at Taikang Tongji COVID-19 Hospital in Wuhan, China, were recruited in the single-center, retrospective study [7]. All patient names were hidden in these tables. The study was approved by the ethical committee of Taikang Tongji COVID-19 Hospital (No. 2020TKTJLL-017). All recruited patients with serum IL-6 level higher than 7.00 pg/mL should be excluded active tuberculosis, bacterial infection, and hepatitis virus B and C infection before TCZ therapy.

### 2.2. Procedures

The data of clinical characteristics, comorbidities, treatments, laboratory results, and clinical outcomes of the patients were obtained from the medical records in detail. COVID-19 was classified into four types, mildly ill, moderately ill, seriously ill, and critically ill, according to the Diagnosis and Treatment Protocol for COVID-19 [7]. The serum levels of IL-6 and C-reactive protein (CRP) were detected before and after TCZ administration. Based on laboratory data in our hospital, the level of IL-6 was defined as elevated when it was higher than 7.00 pg/mL. The CRP was defined as elevated when it was higher than 10.00 mg/L, while the lymphocyte count declined as it was less than 1100/*μ*L. The patients whose laboratory data of IL-6 or CRP or lymphocyte count were completely missed before or after TCZ administration were considered as study withdrawal. The latest laboratory values before TCZ administration were selected as the value before TCZ therapy, and the changes in the value after TCZ administration were observed for one week. The clinical outcome of the patients was evaluated when discharged or died according to the related medical records.

### 2.3. Statistical Analysis

Statistical analysis was done with SPSS, version 13. Data were presented as median (min–max) or as the number and percentage. The Wilcoxon signed-rank test was applied to compare parameters whenever appropriate. A *p* value of less than 0.05 was considered statistically significant.

## 3. Results

### 3.1. The Clinical Characteristics of COVID-19 Patients Treated with TCZ

Thirteen patients (11 males and 2 females) with COVID-19 were included in this study. The characteristics of patients, the use of TCZ, and other anti-inflammatory drugs were summarized in Table 1. The median age (min–max) of these patients was 68 (54–83) years, and the median time from onset to before TCZ therapy was 20 (9–41) days. 7.69% (1/13) of the patients were moderately ill, 61.54% (8/13) of the patients were seriously ill, and 30.77% (4/13) of the patients were critically ill. 84.62% (11/13) of the patients had one or more comorbidities, including hypertension, coronary heart disease, chronic obstructive pulmonary diseases, diabetes, and stroke history. Eleven patients received TCZ administration once, while the other 2 patients received it twice. Two patients received TCZ in combination with methylprednisolone. The dose of TCZ used in patients was 200 mg to 400 mg per time (4–8 mg/kg/per time) (Table 1).

### 3.2. The IL-6 Levels of COVID-19 Patients before and after TCZ Treatment

The IL-6 findings of the 13 patients before and at the first week after TCZ treatment are summarized in Table 2. Elevated serum level of IL-6 is the indication for TCZ therapy in COVID-19 patients [8]. The median level of IL-6 before TCZ administration was 27.91 (7.42–210.90) pg/mL. After starting TCZ therapy, serum IL-6 level of 76.92% (10/13) of the patients tended to spike shortly and then gradually decreased. One patient (No. 9) demonstrated a persistent decrease of IL-6 after TCZ administration. In these 2 critically ill patients who failed the treatment and passed away at the end (No. 1 and No. 10), a persistent and dramatic increase of IL-6 was observed, ranging from 12.84 and 100.4 pg/mL to 2932 and 1308 pg/mL, respectively.

### 3.3. The CRP Levels of COVID-19 Patients before and after TCZ Treatment

The CRP levels of 76.92% (10/13) of the patients were above the normal range before the start of TCZ therapy and gradually decreased after the TCZ treatment. The value of CRP at day 4 after TCZ therapy significantly decreased compared with before TCZ therapy, which dropped from 39.28 (10.82–96.24) mg/L to 4.39 (0.5–7.01) mg/L (*p* < 0.01). Although TCZ had beneficial for relieving inflammatory activity, 2 of them (No. 1 and No. 10) were still dead during the week-long session. In other 11 patients, CRP levels were in the normal range within one week (Table 3).

### 3.4. The Lymphocyte Counts of COVID-19 Patients before and after TCZ Treatment

Lymphocytopenia is a common feature in patients with COVID-19 and might be a critical factor associated with disease severity and mortality [2]. Regarding No. 1 and No. 10, the number of lymphocytes persistently decreased from 330 and 660/*μ*L to 180 and 490/*μ*L at the end, respectively, despite TCZ therapy (Table 4).

### 3.5. The Clinical Outcomes of These Patients after TCZ Treatment

The clinical outcomes of 10 patients were evaluated to be clinical improvement when discharged according to the corresponding medical records. No. 1 and No. 10 died on the 5th or 11th day after TCZ treatment, respectively. No. 13 patient had a clinical outcome of aggravation possible due to inducing severe bacterial infection after TCZ treatment.

### 3.6. The Typical Case Presentation of TCZ Treatment

An 83-year-old man (No. 1) was admitted to a fever clinic on February 16, 2020 (day 1 of illness), with symptoms of intermittent fever, dry cough, and fatigue (Table 5). The CT scan of the local hospital showed multiple patchy shadows in the right lungs, and a nasopharyngeal swab sample was taken. On February 19 (day 4 of illness), the Wuhan Center for Disease Control confirmed by reverse real-time polymerase chain reaction assay that the patient had COVID-19. He was immediately admitted to the isolation ward of our hospital. The initial complete blood count of the patient included white-cell count 4.07 × 10^9^/L and absolute lymphocyte count 0.56 × 10^9^/L. The laboratory test results of the patient during the entire hospitalization were shown in Table 6.

**Figure 1:**
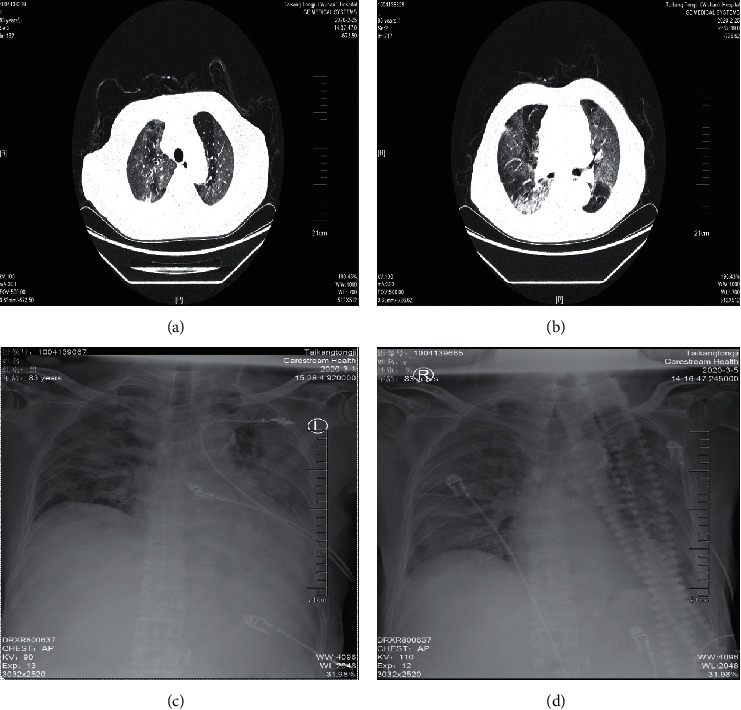
Imaging manifestation of chest CT and X-rays. (a) and (b) showed diffuse interlobular septum thickening in both lungs to form ground-glass opacities and thickening of the bronchial wall. (c) and (d) showed multipatchy shadows and diffused ground-like opacities in both lungs.

He received low flow intranasal oxygen inhalation and was given Lianhuaqingwen capsule (a kind of Chinese herb medicine, four capsules three times daily, orally) and Arbidol (200 mg three times daily, orally) as antiviral therapy subsequently and moxifloxacin (0.4 g once daily, orally) to prevent secondary infection. After receiving medication, he reported that his cough and fatigue slightly improved but reported the feeling of increasing shortness of breath. On day 10 of illness, the arterial blood gas (ABG) analysis showed that pressure of oxygen in arterial blood was 53 mmHg, and pressure of carbon dioxide was 31.3 mmHg, indicating that he suffered from type 1 respiratory failure. The high-resolution-computed tomography of the chest showed diffused ground-like opacities in both lungs (Figures 1(a) and 1(b)). Given the shortness of breath and respiratory failure, methylprednisolone (40 mg twice daily, intravenously) was administered to attenuate lung inflammation. He was diagnosed as a critically ill type of COVID-19 and refused invasive ventilator support in the intensive care unit repeatedly because he suffered from claustrophobia. Therefore, he received high-flow nasal cannula (HFNC) oxygen therapy (85% concentration; flow rate 40 L/min). On day 16 of illness, the patient's symptoms had still not improved, but oxygen saturation remained above 93%. Given the elevated values of IL-6 and C-reactive protein, TCZ (4 mg/kg once, intravenously) was introduced to treat the possible cytokine storm. After receiving the medication, his body temperature reduced from 38.7 to 36.3°C. However, his persistent hypoxemia did not alleviate, multiple patchy shadows on chest radiographs before and after treatment did not improve (Figures 1(c) and 1(d)), and the values of IL-6 swiftly increased from 648.5 pg/ml to 2932.0 pg/ml and the level of procalcitonin elevated quickly followed by empirical anti-infective therapy meropenem plus voriconazole. In the afternoon of day 21 of illness, his hypoxemia and shortness of breath worsened. Despite receiving HFNC oxygen therapy (100% concentration; flow rate 50 L/min), the oxygen pressure value decreased to 27 mmHg, and the patient had a sudden cardiac arrest. He died at 17 : 00 (Beijing time) despite the active rescue.

## 4. Discussion

In the study, the effect of TCZ therapy on COVID-19 patients in the real world was evaluated. Our findings supported that TCZ may have a certain therapeutic effect in the prevention or treatment of cytokine storm induced by COVID-19.

TCZ is a humanized, immunoglobulin G1*κ* (IgG1*κ*) anti-human IL-6 receptor monoclonal antibody approved for the treatment of rheumatoid arthritis, juvenile idiopathic arthritis, and polyarticular juvenile rheumatoid arthritis. It has also been reported to have activity in Castleman disease, Crohn's disease, and steroid refractory chronic graft-versus-host disease [11, 12]. TCZ prevents IL-6 binding to both cell-associated and soluble IL-6 receptors and therefore inhibits both classical and trans-IL-6 signaling [13]. It was also approved by the Food and Drug Administration of America to treat chimeric antigen receptor T cell-induced severe or life-threatening cytokine release syndrome [14]. The dose of TCZ approved for adults with rheumatoid arthritis is 4 to 8 mg/kg every 4 weeks, and the pediatric recommended dose is 8 to 12 mg/kg every 2 to 4 weeks [11, 12]. In China, the dosage of TCZ for treating COVID-19 patients is initially recommended as 4 to 8 mg/kg, if necessary, applied the same dosage repeatedly within 12 hours [7].

The uncontrolled excessive or persistent IL-6 production plays a pathological role in the development of various inflammatory diseases, indicating that IL-6 is a double-edged sword for the host [5]. Dynamic observation of IL-6 levels is also helpful in understanding the progression of COVID-19 and the response to treatment [5]. It was shown that serum IL-6 level of most patients tended to spike shortly and then gradually decreased after starting TCZ therapy. The free serum IL-6 increased because IL-6 receptor-mediated consumption of IL-6 was inhibited by the unavailability of TCZ-free IL-6 receptor. As long as free TCZ was detectable, soluble IL-6 receptor was saturated with TCZ, and IL-6 signaling was completely inhibited [13]. This is the likely explanation for the inhibition of inflammatory activity by TCZ, resulting in improvement of clinical outcomes. Most patients showed clinical improvement regardless of once or twice administration of TCZ, which was different from the results of the other study [8]. This may be a reflection of the powerful anti-inflammatory effect of TCZ. In addition, a persistent and dramatic increase of IL-6 was observed in 2 patients who were finally dead. This reminds us that if the IL-6 level is found to continue to increase during clinical practice, even more than one thousand, it indicates that a cytokine storm has erupted and the patient condition is getting worse, with possible death at the end.

In most patients, the acute phase reactant CRP levels were above the normal range before the start of TCZ therapy and gradually declined after the TCZ treatment. Lymphocytopenia is a common feature in patients with COVID-19 and might be a critical factor associated with disease severity and mortality [2, 4]. No. 1 and No. 10 patients finally died accompanied by the corresponding lymphocyte counts persistently dropping, indicating that we should monitor the changes of lymphocyte count to judge the severity and prognosis of COVID-19.

It is important to note that No. 1 and No. 13 patients had a clinical outcome of aggravation. After TCZ treatment, these patients developed an increased level of blood procalcitonin. The chest CT showed the range of multiple ground-glass changes in both lungs expanded. These suggest that the aggravation of these patient conditions might be related to the use of TCZ. In clinical trials of rheumatoid arthritis, the most common adverse effects and severe adverse effects occurring during TCZ monotherapy or combination therapy were infections and infestations. Serious infections and infestations occurred in 8.5% of TCZ monotherapy recipients and 7.5% of TCZ plus conventional synthetic disease-modifying antirheumatic drug recipients [15]. In a randomized double-blind trial of TCZ in adults with systemic sclerosis, serious infections were found more common in the TCZ group (7 (16%) of 43 patients) than in the placebo group (2 (5%) of 44) [16]. Therefore, we should monitor the changes in procalcitonin level and white blood cell count to judge whether an infection has been induced after TCZ application and intervention as early as possible.

Similarly, at the Zhongfaxincheng campus of Tongji Hospital in Wuhan, three critically ill COVID-19 patients who received only a single dose of TCZ died, while one patient had a clinical outcome of disease aggravation Another eleven patients achieved good results after treatment with TCZ [17]. In conclusion, these are only retrospective observational studies, and there are some limitations, including too few cases, only a self-controlled report before and after without control cases, and some data are incomplete. Furthermore, the treatment duration observed in our study may not be sufficient to make a final conclusion. In a randomized clinical trial of patients with COVID-19 and pneumonia requiring oxygen support, TCZ did not reduce the World Health Organization 10-point Clinical Progression Scale scores lower than 5 at day 4 but might have reduced the risk of needed noninvasive ventilation, mechanical ventilation, or death by day 14 [18]. Therefore, it is necessary to further evaluate the efficacy and safety of TCZ by rigorous randomized controlled trial in the next step.

## Figures and Tables

**Table 1:** The clinical characteristics of COVID-19 patients treated with TCZ.

Case no.	Age	Clinical classification	Comorbidity	Time from onset to before TCZ therapy (days)	TCZ therapy				
					Day 0	Day 1	Day 2	Day 3	Days 4–7
1	83	Critically ill	Hypertension	15	TCZ 480 mg; MP 40 mg	MP 40 mg
2	54	Critically ill	Diabetes	17	TCZ 400 mg	TCZ 400 mg
3	62	Moderately ill	Diabetes CHD	37	TCZ 400 mg
4	83	Seriously ill	Hypertension diabetes	22	TCZ 200 mg
5	71	Seriously ill	None	22	TCZ 400 mg
6	66	Seriously ill	None	9	TCZ 280 mg
7	62	Seriously ill	Stroke history	20	TCZ 400 mg; MP 40 mg	MP 40 mg
8	79	Seriously ill	Hypertension COPD	14	TCZ 200 mg
9	63	Seriously ill	CHD	41	TCZ 200 mg
10	68	Critically ill	COPD	40	TCZ 400 mg	TCZ 400 mg
11	60	Seriously ill	Hypertension diabetes	12	TCZ 400 mg
12	72	Critically ill	Hypertension CHD	10	TCZ 400 mg
13	69	Seriously ill	Hypertension	32	TCZ 400 mg

M: male, F: female. MP: methylprednisolone. CHD: coronary heart disease. COPD: chronic obstructive pulmonary diseases.

**Table 2:** The IL-6 levels of COVID-19 patients before and after TCZ treatment (pg/mL).

Case no.	Before TCZ therapy	After TCZ therapy							Clinical outcomes
		Day 1	Day 2	Day 3	Day 4	Day 5	Day 6	Day 7	
1	12.84	24.40	648.5			2932			Death
2	210.9	339.6	314.2	254.2	92.43	103.5		83.48	Clinical improvement
3	27.91		122.9		101.4				Clinical improvement
4	18.60				146.2				Clinical improvement
5	44.45	370.6			55.29			16.25	Clinical improvement
6	7.42	104.6				110.1			Clinical improvement
7	56.18	36.15		60.99				37.88	Clinical improvement
8	32.01	646.6				521.7		135.5	Clinical improvement
9	57.32				23.30			19.77	Clinical improvement
10	100.4	454.6	1245	1521		1177	940	1308	Death
11	27.90	63.72						84.56	Clinical improvement
12	20.57		84.87		54.87				Clinical improvement
13	9.42	1207		615.9			155.3		Clinical aggravation

The normal value of IL-6 ranges from 0.00 to 7.00 pg/mL.

**Table 3:** The CRP levels of COVID-19 patients before and after TCZ treatment (mg/L).

Case no.	Before TCZ therapy	After TCZ therapy							Clinical outcomes
		Day 1	Day 2	Day 3	Day 4	Day 5	Day 6	Day 7	
1	25.32	17.69	11.52						Death
2	96.24	51.9	25.54	11.56	5.9			0.75	Clinical improvement
3	33.58		10.62		2.87			0.5	Clinical improvement
4	2.94	1.76			0.5				Clinical improvement
5	72.8	46.78			7.01		1.11		Clinical improvement
6	13.66	7.91				1.30		0.5	Clinical improvement
7	31.95	82.93		18.10				0.5	Clinical improvement
8	44.97	42.27				6.01		0.5	Clinical improvement
9	3.56			2.51					Clinical improvement
10	49.5		57.15	32.89					Death
11	6.25	9.59						0.5	Clinical improvement
12	10.82				2.17				Clinical improvement
13	63.76	40.94		0.5	6.92		0.5	0.5	Clinical aggravation

The normal value of CRP ranges from 0.00 to 10.00 mg/L.

**Table 4:** The lymphocyte counts of COVID-19 patients before and after TCZ treatment (/*μ*L).

Case no.	Before TCZ therapy	After TCZ therapy							Clinical outcomes
		Day 1	Day 2	Day 3	Day 4	Day 5	Day 6	Day 7	
1	330	260	240			180			Death
2	680	910	1110	1140	1590			2400	Clinical improvement
3	1220		780		950			1160	Clinical improvement
4	1090	870			890				Clinical improvement
5	1270	950			1740				Clinical improvement
6	780	690				1030		1070	Clinical improvement
7	940	547		1180				1510	Clinical improvement
8	1070	1020				1980			Clinical improvement
9	1030				1020				Clinical improvement
10	660	700	570	640		220	270	490	Death
11	1150	900						1040	Clinical improvement
12	1470		1089		1180			1598	Clinical improvement
13	1280	1420		1580	1690		1670		Clinical aggravation

The normal value of lymphocyte counts ranges from 1100 to 3200/*μ*L.

**Table 5:** Timeline of disease course according to days from initial presentation of illness and days from hospital admission, from February 16 to March 7, 2020. SARS-CoV-2 = severe acute respiratory syndrome coronavirus 2.

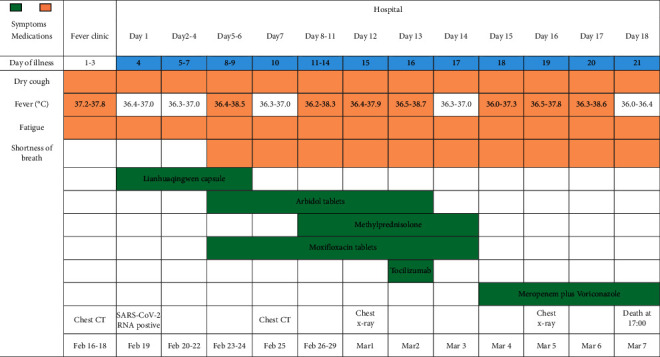

**Table 6:** Clinical laboratory tests of the patient.

Measure	Reference range	Illness day 4 hospital day 1	Illness day 9 hospital day 6	Illness day 10 hospital day 7	Illness day 12 hospital day 9	Illness day 15 hospital day 12	Illness day 16 hospital day 13	Illness day 17 hospital day 14	Illness day 20 hospital day 17	Illness day 21 hospital day 18
*Complete blood count*										
White-cell count (×10^9^/L)	3.5–9.5	4.07	4.16	3.6	5.39	13.21§	12.06§	13.98§	14.00§	
Absolute neutrophil count (×10^9^/L)	1.8–6.3	3.25	3.38	3.21	4.86	12.28§	11.50§	13.47§	13.52§	
Absolute lymphocyte count (×10^9^/L)	1.1–3.2	0.56‡	0.56‡	0.29‡	0.35‡	0.33‡	0.26‡	0.24‡	0.18‡	
Absolute monocyte count (×10^9^/L)	0.1–0.6	0.25	0.22	0.10	0.17	0.56	0.25	0.19	0.19	
Red-cell count (×10^12^/L)	3.8–5.1	5.19	4.59	4.59	4.75	5.01	4.84	4.87	4.10	
Hemoglobin (g/L)	115–150	153	140	136	142	149	144	146	124	
Platelet count (×10^9^/L)	125–350	107	93‡	104‡	131	181	121	100	71‡	

*Biochemical test*										
Total protein (g/L)	65–85			53.62	60.04	61.65	59.19	56.89	48.52	
Albumin (g/L)	35–55			28.76‡	30.25‡	26.70‡	25.54‡	23.22‡	25.90‡	
Globulin (g/L)	20–40			24.86	29.79	34.95	33.65	33.67	22.62	
Alanine aminotransferase (ALT) (U/L)	7–45			20.92	34.97	59.99§	54.47§	46.85§	20.07	
Glutamyl transpeptidase (GGT) (U/L)	5–50			142.33§	165.87§	198.86§	199.83§	186§	126.28§	
Lactate dehydrogenase (LDH) (U/L)	140–271			331.83§	424.24§	486.32§	541.48§	619.78§	749.45§	
Urea (mmol/L)	2.8–7.2			6.22	9.77§	8.91§	9.17§	10.37§	15.18§	
Creatinine (umol/L)	49–90			74.34	84.02	83.72	86.00	77.28	77.28	
Sodium (mmol/L)	137–147			131.60‡	134.60‡	137.70	137.10	136.60‡	142.70	
Potassium (mmol/L)	3.5–5.3			4.00	3.76	3.91	4.07	4.12	4.03	
Chloride (mmol/L)	96–108			96.20	98.70	102.90	102.70	100.30	107.60	

*Arterial blood gas (ABG) analysis*										
Fracture of inspired oxygen (FiO2)				33%	85%	85%	85%	85%	85%	100%
Potential of hydrogen (PH)	7.35–7.45			7.457§	7.452§	7.498§	7.478§	7.512§	7.474§	7.429
Pressure of oxygen in arterial blood (mmHg)	90–100			53‡	73‡	55‡	61‡	87‡	49‡	27‡
Pressure of carbon dioxide in arterial blood (mmHg)	35–45			31.3‡	30.7‡	30.6‡	29.6‡	32.4‡	36.6	42.1
Base excess (mmol/L)	−3.0-3.0			−2.0	−1.0	1.0	0.0	4.0§	3.0	3.0
HCO3 (mmol/L)	22–27			22.1	22.0	23.8	21.9	25.9	26.9	29.0§

*Coagulation profile*										
Prothrombin time (sec)	10.2–14.3			11.7		12.5		15.8	21.4	
International normalized ratio	0.8–1.2			1.06		1.16		1.65	1.98	
Fibrinogen C (mg/dL)	2.0–4.0			409		230		154	102	
D-dimer (ng/mL)	0–0.5			250			12397	15216		
CRP (mg/dL)	0–0.5	1.94			25.32§		17.69§	11.52§		
Procalcitonin (ng/mL)	0.00–0.05			0.216§	0.226§	0.117§	0.176§	0.188§	3.680§	
Interleukin-6 (pg/mL)	0–7			17.88§	7.34§	12.84§	24.4§	648.5§	2932§	
Blood culture						Negative				
Hepatitis B surface antigen (HBsAg)	0–1					0.429				
Hepatitis C virus (HCV) antibody	0–1					0.039				
Human immunodeficiency virus (HIV) antibody	0–1					0.20				

^‡^The value in the patient was below normal. ^§^The value in the patient was above normal.

## Data Availability

The data used to support the findings of this study are included within the paper.
